# Early-Life Exposure to Lipopolysaccharide Induces Persistent Changes in Gene Expression Profiles in the Liver and Spleen of Female FVB/N Mice

**DOI:** 10.3390/vetsci10070445

**Published:** 2023-07-08

**Authors:** Elda Dervishi, Dagnachew Hailemariam, Seyed Ali Goldansaz, Burim N. Ametaj

**Affiliations:** Department of Agricultural Food, and Nutritional Science, University of Alberta, Edmonton, AB T6G 2P5, Canada; dervishi@ualberta.ca (E.D.); hailemar@ualberta.ca (D.H.); goldansaz@ualberta.ca (S.A.G.)

**Keywords:** mice, LPS, gene expression, liver, spleen

## Abstract

**Simple Summary:**

This study aimed to examine the impact of lipopolysaccharide (LPS) on the gene expression patterns of insulin signaling, innate immunity, and adaptive immunity in the liver and spleen of mice. The findings demonstrate that prolonged and continuous exposure to endotoxemia during early life (at 35 days of age) can initiate molecular mechanisms that result in lasting alterations in gene expression associated with inflammation and adaptive immunity. These changes may contribute to the development of various chronic inflammatory diseases in the liver and spleen. The results indicate that exposing mice to chronic and continuous endotoxemia during early life (at 35 days old) for a duration of 6 weeks can trigger molecular mechanisms that induce lasting changes in gene expression. These changes are associated with inflammation and adaptive immunity and may play a role in the pathobiology of diverse chronic inflammatory diseases affecting the liver and spleen.

**Abstract:**

The objective of this study was to investigate how subcutaneous (sc) lipopolysaccharide (LPS) administration affects the gene expression profiles of insulin signaling as well as innate and adaptive immunity genes in mouse livers and spleens. FVB/N female mice were randomly assigned to one of two treatment groups at 5 weeks of age: (1) a six-week subcutaneous injection of saline at 11 μL/h (control—CON), or (2) a six-week subcutaneous injection of LPS from *Escherichia coli* 0111:B4 at 0.1 μg/g body weight at 11 μL/h. At 106 weeks (i.e., 742 days) after the last treatment, mice were euthanized. Following euthanasia, liver and spleen samples were collected, snap frozen, and stored at −80 °C until gene expression profiling. LPS upregulated nine genes in the liver, according to the findings (*Pparg, Frs3, Kras, Raf1, Gsk3b, Rras2, Hk2, Pik3r2,* and *Myd88*). With a 4.18-fold increase over the CON group, *Pparg* was the most up-regulated gene in the liver. Based on the annotation cluster analysis, LPS treatment upregulated liver genes which are involved in pathways associated with hepatic steatosis, B- and T-cell receptor signaling, chemokine signaling, as well as other types of cancers such as endometrial cancer, prostate cancer, and colorectal cancer. LPS increased the spleen expression of *Ccl11, Ccl25, Il6, Cxcl5, Pparg, Tlr4, Nos2, Cxcl11, Il1a, Ccl17,* and *Fcgr3,* all of which are involved in innate and adaptive immune responses and the regulation of cytokine production. Furthermore, functional analysis revealed that cytokine–cytokine receptor interaction and chemokine signaling pathways were the most enriched in LPS-treated mice spleen tissue. Our findings support the notion that early-life LPS exposure can result in long-term changes in gene expression profiling in the liver and spleen tissues of FVB/N female mice.

## 1. Introduction

Lipopolysaccharide (LPS), also known as endotoxin, is the major constituent of Gram-negative bacteria’s outer membrane, accounting for three-quarters of the outer membrane [1]. Lipopolysaccharide is composed of three components: lipid A, a core polysaccharide, and an O-specific polysaccharide chain [2]. Lipopolysaccharide can enter the host’s systemic circulation and cause inflammation when it is released from bacterial membranes during growth, death, or lysis [3]. Many different types of host cells interact with LPS, including antigen-presenting cells like macrophages and dendritic cells [4,5,6].

Both macrophages and dendritic cells express pattern recognition receptors such as Toll-like receptor 4 (TLR4) to recognize microbial LPS [6,7]. TLR4 activation by LPS results in the release of tumor necrosis factor (TNF), interleukin 1 (IL-1), IL-6, IL-8, and IL-10 [7,8,9,10]. Tumor necrosis factor, IL-1, and IL-6 are proinflammatory cytokines that initiate and control local and systemic inflammatory responses, as well as innate and adaptive immune responses. A prolonged immune response to LPS, on the other hand, has serious consequences for specific organs and the host itself. Endotoxemia has been shown to cause systemic inflammation, which has been linked to obesity, diabetes, insulin resistance, and an increased risk of heart disease [11,12,13,14]. Endotoxin binding to TLR-4 on vascular endothelial cells has also been proposed to promote atherosclerotic plaque activation [15,16,17]. Endotoxin has also been shown to increase pancreatic cancer cell invasiveness via the TLR4/MyD88 signaling pathway [18]. Beta-1 integrin-mediated cell adhesion and liver metastasis are also increased by LPS [19].

Furthermore, there is compelling evidence that endotoxin contributes to the onset and progression of alcoholic liver disease (ALD). Endotoxin in the intestinal lumen enters the bloodstream, causing cytokine production from Kupffer cells and initiating the process of liver inflammation, inflammatory cell infiltration, and fibrosis [20,21].

The major mediators of the immune response and systemic inflammatory processes are acute-phase proteins and cytokines produced by liver and spleen [22,23,24]. Understanding the impact of chronic LPS exposure on gene expression patterns in different immune system organs is therefore critical.

We hypothesized that chronic sc administration of LPS early in life would result in long-term differential gene expression changes in the liver and spleen of FVB/N female mice. As a result, the objectives of this study were to follow selected gene expression profiles in the liver and spleens of FVB/N female mice treated subcutaneously with LPS from *Escherichia coli* 0111:B4 for 6 weeks starting at 5 weeks of age and euthanized 106 weeks later.

## 2. Materials and Methods

### 2.1. Animals and Experimental Design

The Animal Care and Use Committee, Health Sciences, at the University of Alberta approved all methods used during the study in accordance with the Canadian Council on Animal Care [25]. 

The FVB/N female mice were housed in groups of five per cage, with food and water available at all times and a 12 h light/dark cycle. After one week of acclimatization, five-week-old FVB/N mice (*n* = 5) were randomly assigned to one of two treatment groups: (1) six weeks of saline administration at 11 μL/h, or (2) six weeks of *Escherichia coli* 0111:B4 LPS injection at 0.1 μg/g BW at 11 μL/h.

To avoid the need for repeated injection schedules and frequent animal handling, ALZET^®^ osmotic mini pumps (ALZET, Cupertino, CA, USA) were used to administer saline and LPS sc to both groups. ALZET^®^ mini-osmotic pumps were inserted subcutaneously (sc) to simulate continuous and minimal oral entry of LPS into the body [26]. During the sc infusion, the manufacturer’s recommended procedure was followed. Mice were anesthetized using isoflurane (Baxter Corporation, Mississauga, ON, Canada). Once the mouse became unresponsive to tail pinching, an anesthetic machine was used to administer a low and continuous flow of isoflurane and oxygen (Matrx by Midmark Corporation, Versailles, OH, USA). After shaving and disinfecting a small area on the back of the mouse, a cut was made with sterile surgical scissors. Sutures were used to close the opening after the ALZET^®^ mini-osmotic pumps were inserted (ALZET Osmotic Pumps, Cupertino, CA, USA). The same procedure was repeated after 6 weeks to remove the empty pumps and close the skin opening with sutures [26]. 

### 2.2. Euthanasia

Five mice (n = 5) were euthanized 106 weeks (742 days) after the treatment. Isoflurane (Matrx by Midmark Corporation, Versailles, OH, USA) gas overdose was used to anaesthetize mice [26]. After assessing their reflexes and confirming that they were no longer feeling pain, euthanasia was performed by drawing all of their blood through a cardiac puncture. Following euthanasia, the whole liver and spleen were collected, snap frozen and stored at −80 °C. Additionally, beginning with the administration of the treatment at 6 weeks of age, the body weight of each individual mouse in each group was assessed every month. 

### 2.3. Total RNA Isolation and First Strand cDNA Synthesis

Following the manufacturer’s instructions, total RNA was extracted from liver and spleen tissues (n = 3) using the SV total RNA Isolation System (Promega Corporation, Madison, WI, USA). A 30 mg sample was collected for RNA isolation after samples were thawed at room temperature. After that, samples were lysed in an autoclaved tube containing 175 μL of RNA lysis buffer (RLA + BME). Samples were cooked at 70 °C for 3 min after 350 μL of RNA dilution buffer (RDA) had been added. After that, samples were centrifuged at 14,000× g for 10 min (model 5430R, Eppendorf, Hamburg, Germany). Two hundred microliters (95%) of ethanol (Commercial Alcohol, Winnipeg, MB, Canada) was added after the clear lysate had been collected into a new 5 mL tube (Promega, Madison, WI, USA). Lysate was cleaned with RNA wash solution after being transferred to the spin basket assembly (Promega, Madison, WI, USA) and centrifuged for 1 min. DNase I was then used to perform on-column DNA digestion in order to eliminate any DNA contamination.

Following additional washing with washing buffers, 100 μL of nuclease-free water was used to elute the total RNA. The purity and concentration of each RNA sample was determined using Nanodrop 8000 instrument (based on measurements at 260 and 280 nm) (peqLab Biotechnologies GmbH, Erlangen, Germany). A cut-off of 1.8 for the A 260/A 280 ratio was employed to assess RNA integrity.

The RT2 miRNA first Strand kit (SABiosciences, Frederick, MD, USA) was used to synthesize cDNA, following the manufacturer’s instructions. First, 0.65 μg of total RNA and 2 μL of 5× gDNA elimination buffer were combined to create the first genomic DNA elimination mix. The final volume was adjusted to 10 mL by adding ddH2O. The mixture was gently stirred using a pipette (Eppendorf, Hamburg, Germany), briefly centrifuged (Eppendorf, Hamburg, Germany), and incubated at 42 °C for 5 min. Thereafter, a 10 μL RT cocktail was made for each sample by mixing 4 μL of 5× RT buffer 3 (BC3), 1 μL of primer and external control mix (P2), 2 μL of RT enzyme mix 3 (RE3), and 3 μL of ddH_2_O. Then, 10 μL of the RT cocktail was added to 10 μL of each of the genomic DNA removal mixtures. The mixture was gently blended using a pipettor, then incubated at 42 °C for 15 min. The reaction was stopped by heating the mixture to 95 °C for 5 min. Each 20 μL cDNA synthesis reaction was then administered 91 μL of ddH2O and stored at −20 °C until analysis.

### 2.4. Quantitative PCR (qPCR) Assay

The gene expression profiling in liver and spleen tissues was determined using qPCR on a StepOnePlus ABI Prism platform (Applied Biosystems, Burlington, ON, Canada). To assess gene expression in the liver, the Mice Insulin Signaling Pathway Kit and the Innate and Adaptive Immunity PCR Array Kit were used (Qiagen, Mississauga, ON, Canada). The Insulin Signaling Pathway Kit examines 84 genes that are involved in cell growth and differentiation, transcription factors, glucose, lipid, and protein metabolism, insulin receptor-associated proteins, and the PI-3 kinase and MAPK pathways. The Innate and Adaptive Immunity kit detects 84 genes associated with IL-1R/TLR members, host defense against bacteria, innate immune response, and septic shock genes. Both PCR array kits included specific primers for 84 genes, a control for genomic DNA contamination (GDC), a control for reverse transcription (RTC), and a positive PCR control. At the end stage, spleen samples were collected, and gene expression was evaluated using a RT2 PCR array. The 96-well custom RT2-PCR array was used to quantify 84 inflammation-related genes, 5 housekeeping genes (B2M, Actb, Gapdh, Gusb, and HSP90ab1), 3 reverse transcription controls (RTC), 3 positive PCR controls, and mouse genomic DNA contamination controls. An Excel spreadsheet containing the mouse gene symbol and ResSeq number was sent to SAbioscience for manufacturing (SABiosciences, Frederick, MD, USA). 

Prior to real-time PCR profiling, each of the 20 μL first strand cDNA products from all biological replicates from the treatment and negative control groups received 91 μL of DNase/RNase-free water. Then, 102 μL of diluted cDNA template, 1350 μL of RT2 SYBR Green PCR master mix, and 1248 μL of DNase/RNase-free water were combined to create a PCR master mix. Twenty-five microliters of this mixture was added to each well of the 96-well plate, which contained sequence-specific primer sets for each gene and their respective controls. After a brief centrifugation (Eppendorf, Hamburg, Germany), the plate was loaded onto the StepOnePlus Real-Time PCR System (Applied Biosystems, Darmstadt, Germany) and run with a thermal program of initial heating at 95 °C for 10 min, followed by 40 cycles of 95 °C for 15 s and 60 °C for 1 min. A melting curve generated at the end of the PCR protocol was used to control the specificity of the amplification.

### 2.5. Statistical Analysis

The ∆∆CT method from the PCR array data analysis web portal “http:///www.sabiosciences.com/pcrarraydataanalysis.php (accessed on 14 October 2015)” was used to analyze the fold change in the mRNA expression of selected genes included in the custom PCR array. For each gene in the custom PCR array, the software calculated the average threshold cycle values for biological replicates from both the treatment and control groups. All genes with a ct value greater than 35 and those that were undetected were excluded from the analysis. The data were normalized by adjusting all comparative threshold (ct) values for the average ct value of the array’s endogenous controls [26].

Gene expression data were analyzed with t-tests and treatment was included as a fixed factor. Fold change values greater than 1.5 and *p* ≤ 0.05 were used as cut-off values to identify genes that were differentially expressed in the treatment groups versus the saline-treated negative control group. MetaboAnalyst “https://www.metaboanalyst.ca (accessed on 25 April 2023)” was used to perform cluster analysis for gene expression [27]. Each sample began as a separate cluster in hierarchical cluster analysis, and the algorithm proceeded to combine them until all samples belong to one cluster. When performing hierarchical clustering, two parameters were considered—the first was a similarity measure, and the second was a clustering algorithm. The Euclidian and Ward clustering algorithms were used to measure distance [27]. 

The Functional Annotation Cluster (FAC) tool based on the Gene Ontology (GO) annotation function was used to determine pathways and processes of major biological significance and importance using the Database for Annotation, Visualization, and Integrated Discovery (DAVID; https://david.ncifcrf.gov/tools.jsp (accessed on 25 April 2023)) v6.7b [28]. The gene lists obtained after statistical analysis were subjected to a DAVID FAC analysis. The medium stringency EASE score parameters were selected to show confident enrichment scores of functional significance and importance of the investigated pathways and processes. As a threshold for cluster significance, an enrichment score of 1.3 was used [29]. In addition, the ClueGo plug-in and Cytoscape program [30,31] were used visualize the differentially expressed genes in the liver and spleen of mice according to the pathways in which they are involved. We used the Mus musculus database and KEGG pathways to develop a pathway network [30].

## 3. Results

### 3.1. Weight and Gene Expression Profiling in the Liver and Spleen Tissues

Overall, the treatment did not have a significant effect on the weight of the mice (*p* = 0.45). However, there was a significant effect observed for the week factor (*p* < 0.001), indicating that the age of the mice had a notable influence. This is further illustrated in Figure S1. The results of gene expression profiles in the liver and spleen are shown in Table 1 and Table 2, respectively. A total of seven genes were significantly differentially expressed in the liver of mice that received subcutaneous administration of LPS. Six genes (*Pparg*, *Frs3*, *Kras*, *Raf1*, *Gsk3b*, and *Rras2*) were up-regulated in the liver. The most up-regulated gene in the liver tissue was *Pparg*, with a 4.18-fold increase compared to the CON group. In addition, there were tendencies for overexpression of *Hk*2 (*p* = 0.08), *Pik3r2* (*p* = 0.06), *Myd88* (*p* = 0.07), and *Nfkbia* (*p* = 0.07) in the LPS group. In addition, *Cxcl10* was significantly down-regulated, decreasing by 3.6-fold compared to the CON group. Other genes like *Ptprf, Acaca*, *Ccr5*, *Irf3, Irf7, Lyz2, Mbl2*, and *Stat1* tended to be down-regulated (see Table 1 for *p* values).

The results also showed that LPS in the spleen significantly altered the expression of 22 genes (*p* < 0.05). Twelve genes were up-regulated (*Ccl25*, *Il6*, *Ccl17*, *Pparg*, *Ccl11*, *Prnd*, *Cxcl5, Il1a, Cxcl11, Nos2, Tlr4*, and *Fcgr3*) and ten genes (*Fyn, Grn, Lyz2, H2-k1, Fcgr2b, Egr1, Notch1, Rtp4, Ifi27i2a*, and *Ache*) were down-regulated (*p* < 0.05). In addition, there were tendencies for the down-regulation of *Apoe* (*p* = 0.06), *Gbp4* (*p* = 0.07), and *Atp1b1* (*p* = 0.08) in the spleen (Table 2). The most up-regulated gene in the spleen was *Fcgr3* (Fc fragment of Ig, low affinity Il1b, receptor) with a 18.23-fold increase compared to the CON group of mice. *Lyz2* (Lysozyme 2) and *Ifi27i2a* (interferon, alpha-inducible protein 27 like 2A) were the most down-regulated genes, with −11.2- and −8.94-fold changes, respectively, in the LPS-treated group compared to the control (Table 2).

**Figure 1:**
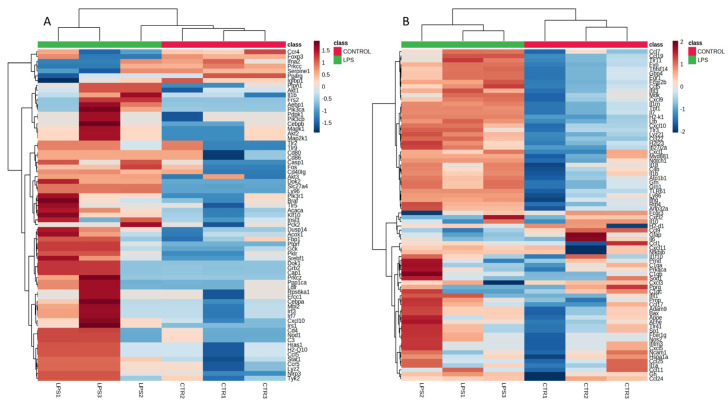
Hierarchical cluster analysis of gene expression in mouse (**A**) livers and (**B**) spleens.

Two genes, *Pparg* and *Lyz2*, were differentially expressed in both the liver and spleen. Both organs exhibited an increased expression for *Pparg* and a decrease in the expression of *Lyz2*. A heatmap of gene expression of the control and LPS group in the liver and spleen is shown in Figure 1a,b, respectively.

### 3.2. Functional Annotation Analysis

The results of DAVID functional annotation clustering (FAC) of differentially expressed genes in the liver tissue showed that the most enriched pathway clusters, with an enrichment score of 2.67, was ‘hepatitis C, followed by ‘chemokine signaling pathway’, ‘endometrial cancer’, ‘prolactin signaling pathway’, ‘EGFR tyrosine kinase inhibitor resistance’, and ‘B-cell receptor signaling pathway’. This pathway involved the genes *Kras*, *Gsk3b*, *Raf1*, *Nfkbia*, and *Pik3r2* (Table 3). In addition, several disease pathways including the ‘endometrial cancer, ‘colorectal cancer’, ‘ErbB signaling’, ‘breast cancer’, and ‘gastric cancer’ pathways with *Kras*, *Gsk3b*, and *Raf1* genes were significantly enriched (Table 3).

The results of FAC of differentially expressed genes in the spleen revealed that the most enriched pathway cluster had an enrichment score of 4.25, followed by a second cluster with a score of 3.07. The top five enriched biological processes were the ‘inflammatory response’ with 10 genes (*Ccl11*, *Ccl17*, *Ccl25*, *Cxcl11*, *Cxcl5*, *Il1a*, *Il6*, *Nos2*, *Pparg*, *Tlr4*), ‘neutrophil chemotaxis’ with 6 genes (*Fgr*, *Ccl11*, *Ccl17*, *Ccl25*, *Cscl11*, *Cxcl5*), ‘positive regulation of ERK1 and ERK2 cascade’ with 7 genes (*Ccl11*, *Ccl17*, *Ccl25*, *Il1a*, *Il6*, *Notch1*, *Tlr4*), ‘cytokine activity’ with 7 genes (*Ccl11*, *Ccl17*, *Ccl25*, *Cxcl5*, *Grn*, *Il1a*, *Il6*) and ‘chemokine activity’ with 5 genes (*Ccl11*, *Ccl17*, *Ccl25*, *Cxcl11*, *Cxcl5*; Table 4). In addition, the results of DAVID FAC showed that ‘viral protein interaction with cytokine and cytokine receptor’ with six genes (*Ccl11*, *Ccl17*, *Ccl25*, *Cxcl11*, *Cxcl5*, *Il6*), ‘chemokine receptors bind chemokines’ with five genes (*Ccl11*, *Ccl17*, *Ccl25*, *Cxcl11*, *Cxcl5*), and ‘cytokine-cytokine receptor interaction’ with seven genes (*Ccl11*, *Ccl17*, *Ccl25*, *Cxcl11*, *Cxcl5*, *Il1a*, *Il6*) were the most enriched pathways in the spleen tissue in the LPS-treated mice (Table 4).

**Figure 2:**
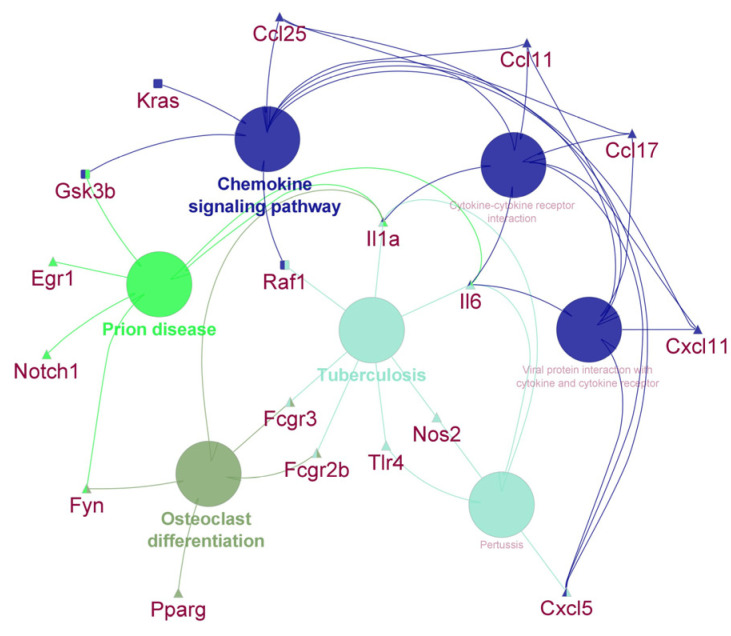
Gene network of differentially expressed genes in the liver and spleen of mice treated with subcutaneous LPS. Genes that are differentially expressed in the liver are represented by a rectangle shape and genes that are differentially expressed in the spleen are represented by a triangle shape. Functionally grouped networks with terms as nodes are linked based on their kappa score level (0.4).

A visualization of the differentially expressed genes in the liver and spleen of mice according to the pathways in which they are involved is shown in Figure 2. The chemokine signaling pathway, cytokine–cytokine receptor interaction and viral protein interaction with cytokines and cytokine receptors were significantly enriched. Furthermore, the differentially expressed genes participate in prion disease and tuberculosis.

## 4. Discussion

LPS injected into the bloodstream or intraperitoneally has been shown to be removed from circulation by monocytes, macrophages, and neutrophils in the systemic circulation, as well as resident macrophages in the liver and spleen [32]. The liver contains nearly 80% of all resident macrophages in the body, making it a critical organ for removing and detoxifying pathogen-borne antigens such as LPS [33]. 

In a related paper, we showed that early-life subcutaneous LPS administration in the same mice resulted in long-term changes in gene expression profiling in the brain 106 weeks later [26]. In this study, we hypothesized that 6 weeks of subcutaneous LPS administration at the age of 35 days would result in long-term changes in the gene expression profiles in the liver and spleen of mice 106 weeks after treatment. 

Indeed, we discovered that mice exposed to LPS at a young age displayed long-term changes in their gene expression profiles in both the liver and spleen. One hundred and six weeks after LPS administration, eight genes (*Pparg, Frs3, Kras, Raf1, Gsk3b, Rras2, Hk2*, and *Pik3r2*) were up-regulated in the livers of mice. When compared to the control group, *Pparg* was the most up-regulated gene in the liver, with a 4.18-fold increase. PPARG binds to compounds that promote the proliferation of peroxisomes, which are liver organelles that aid in the oxidation of fatty acids. Furthermore, *Pparg* regulates energy metabolism, adipocyte differentiation, glucose homeostasis, and the promotion of lipid storage, inducing fatty liver [34]. PPARG, as an anti-inflammatory protein, is important in regulating immune responses during inflammation [35,36,37]. In another study, LPS was shown to downregulate *Pparg* expression in macrophages and in the liver of rats during sepsis via an increase in TNF release [38,39]. The difference can be explained by the fact that the authors of that study measured *Pparg* expression in an animal sepsis model 10 h after administering a single higher dose of LPS. However, in our study, we looked at the long-term effects of a low dose of LPS administered chronically and continuously on *Pparg* expression. 

Intriguingly, genes related to TLR and the immune response pathway were down-regulated in the liver in our study. Because the *Pparg* product is an anti-inflammatory protein, its up-regulation may have contributed to the down-regulation of pro-inflammatory responses in the liver. It is hypothesized that the up-regulation of *Pparg* may have suppressed the activation of liver-resident macrophages (i.e., Kupffer cells) by down-regulating the expression of *Irf7, Irf3, Stat1, Cxcl10, Mbl2, Lyz2*, and *Ccr5*. The mechanisms by which the *Pparg* product suppresses genes involved in the TLR pathway and defense response (i.e., innate immunity) warrant further investigation. 

Regarding fatty liver, *Pparg* overexpression has been observed in several animal models of obesity and diabetes [40,41]. The liver regulates lipid homeostasis by controlling lipid uptake from the circulatory system, de novo synthesis, and the delivery of synthesized lipids to peripheral tissues as very low-density lipoprotein [42]. We previously proposed that LPS contributes to hepatic steatosis by binding to TG-rich lipoproteins and allowing them to be removed from the systemic circulation more quickly by liver hepatocytes [43]. These new findings support the hypothesis that bacterial LPS also contributes to fatty liver via the stimulation of *Pparg* overexpression. Indeed, *Pparg* has been shown to play a direct role in TG and lipid droplet formation in vivo [44,45].

Annotation cluster analysis revealed that up-regulated liver genes like *Kras, Rras2, Raf1, Frs3, Gsk3b, Hk2*, and *Pik3r2* are involved in the ‘B-cell receptor signaling pathway’, the ‘T-cell receptor signaling pathway’, and the ‘chemokine signaling pathway’. The liver is home to a variety of immune cells, including Kupfer cells, natural killer (NK) cells, and T and B lymphocytes [46]. Based on our findings, it is evident that despite the administration of LPS at a significantly low concentration for a duration of 6 weeks starting at the age of 35 days, long-lasting alterations were observed in the expression of genes associated with the ‘B-cell receptor signaling pathway’, the ‘T-cell receptor signaling pathway’, and the ‘chemokine signaling pathway’.

LPS treatment induced cancer-related genes in the liver, including *Kras, Rras2*, and *Raf1*. KRAS is required for normal cell signaling, growth, proliferation, and apoptosis, and mutations in this gene have been linked to a variety of human cancers [47,48]. Somatic activation of the *Kras* oncogene causes lung cancer to develop early in mice [49], and its transcription levels have been linked to a variety of cancers, including cystadenocarcinoma and prostate cancer [50,51]. *Rras2* has been linked to ovarian cancer and chronic lymphocytic leukemia [52,53]. Furthermore, *Raf1* has been linked to tumors of the parotid gland, stomach cancer, and renal cell carcinoma [54,55,56]. According to the annotation cluster analysis, upregulation of three genes, namely *Kras*, *Gsk3b*, and *Raf1*, was observed in LPS-treated mice. These genes are associated with pathways related to ‘endometrial cancer,’ ‘colorectal cancer,’ and ‘prostate cancer’. 

Jiang et al. [57] reported that endotoxin levels in human blood increased significantly with the progression of liver cancer, with the highest level found in the advanced liver cancer group. Another study, Liu et al. [58], concluded that LPS in the chronic liver inflammation microenvironment may play a role in hepatocarcinogenesis by regulating the plastic potential of hepatic progenitor cells. To the best of our knowledge, this is the first study to show that chronic LPS administration early in life upregulates genes associated with cancer pathways in mouse liver tissue nearly two years later.

The up-regulation of genes *Gsk3b* (glycogen synthase kinase-3), *Hk2* (hexokinase 2), and *Pik3r2* (phosphatidylinositol 3-kinase regulatory subunit beta) in the liver of mice treated with LPS is important for the regulation of the activities of both innate and adaptive immune cells during inflammation and disease. GSK3, for example, is involved in the production of pro-inflammatory cytokines in response to TLR stimulation [59]. When pathogens are detected by the innate immune system, inflammation is initiated. Bacteria are phagocytosed by both resident macrophages and infiltrating neutrophils using a variety of cell surface receptors. To kill engulfed bacteria, neutrophils activate an intracellular NADPH oxidase, which produces reactive oxygen species (ROS). Each of these processes necessitates the activation of class I Pi3k [60]. GSK3 deficiency caused by lithium or other *Gsk3* inhibitors, or molecular manipulation, decreases proinflammatory cytokine production by TLR-stimulated monocytes by 67–90% [59]. Moreover, Hk2 supports neoplastic growth in glioblastoma multiforme [61], provides chemoresistance to epithelial cells of ovarian cancer [62], and initiates and maintains lung and breast cancer tumors in lung and breast mouse models, and its inhibition confers therapeutic effects [63].

The spleen is also considered to be an important organ in the removal of LPS from systemic circulation. Indeed, LPS administered intravenously to rats was detected in decreasing concentrations in spleen macrophages between 24 h and a week after administration [64]. Groeneveld and van Rooijen [65] previously reported the distribution of intravenously administered radiolabeled LPS in the spleen of mice. The majority of the LPS was accumulated in macrophages in the splenic marginal zone (MZ). Furthermore, LPS reduced MZ macrophages and eliminated lymphocytes from the marginal zone. Extracellular dendritic localization of LPS in the central parts of spleen follicles, on the other hand, has been reported. These findings are significant because they show that intravenous LPS is captured by splenic immune cells, primarily resident macrophages, and dendritic cells.

According to gene expression profiling in our study, long-term subcutaneous administration of LPS in female mice up-regulated 12 genes and down-regulated 11 genes in the spleen tissue. ‘Inflammatory response’ was the most enriched GO (gene ontology) term, and the most up-regulated pathways in the spleen were ‘viral protein interaction with cytokines and cytokine receptors’, ‘chemokine receptors bind chemokines’ and ‘cytokine–cytokine receptor interaction’ with the genes *Ccl11, Ccl17, Ccl25, Cxcl5, Cxcl11*, *Il6* and *Il-1a*. The first five genes encode critical chemokines, while the remaining two genes encode two key proinflammatory cytokines, interleukin (IL)-6 and IL-1a. Chemokines are a large family of small cytokines that can interact with chemokine receptors, which control immune cell residence and migration and are involved in immunoregulatory and inflammatory processes.

The *Ccl11* gene is up-regulated in response to an increase in the concentration of C-C motif chemokine 11, also known as eotaxin. CCL11 has the highest affinity for the C-C chemokine receptor CCR3, as well as CCR2 and CCR5 [66,67]. It has been reported that CCL11 induces the migration of several types of leukocytes, including eosinophils, basophils, macrophages, and dendritic cells, via interaction with CCR3 [68,69,70,71]. Earlier studies have shown that *Ccl11* is activated by pro-inflammatory cytokines such as tumor necrosis factor (TNF) [72,73,74]. CCL11 is also a strong inducer of eosinophil chemotaxis, which results in eosinophil migration in vitro and accumulation in vivo. A recent study by Xu et al. [75] found that a significant number of eosinophils were recruited to the liver during experimental liver failure in mice, compared to very few eosinophils in normal livers. Plötz et al. [76] discovered that LPS stimulates TNF secretion from eosinophils as well as LPS uptake via the CD14 pathway. There is an increase in the number of eosinophils in the blood circulation in patients who have had a splenectomy [77], indicating the importance of the spleen in controlling the number of eosinophils. Eosinophils are highly active inflammatory cells that produce and secrete a wide range of mediators leading to cytotoxic activity and tissue damage [78,79].

Overexpression of *Ccl17* is intriguing because it interacts with a high-affinity receptor found on T cells and, to a lesser extent, monocytes. This is a chemokine that plays a role in both innate and adaptive immune regulation. CCL17 attracts both natural killer cells and dendritic cells. This chemokine is also important because it attracts Th2 cells and suppresses type 1 innate immune responses while stimulating type 2 immune responses [80]. While type 1 immune responses are characterized by the activation of M1 macrophages associated with phagocytic activity, type 2 immune responses are associated with the activation of M2 macrophages associated with humoral immunity [80]. It is likely that early-life parenteral LPS treatment of mice resulted in long-term changes in the expression of chemokines that promote humoral immune responses to blood-borne LPS. 

C-C chemokine ligand 25 (*Ccl25*) was another overexpressed gene in LPS-treated mice. C-C chemokine receptor 9 (CCR9) is the receptor for CCL25. Because this chemokine is secreted by only a few cell populations, CCL25 synthesis is tightly controlled. *Ccl25* mRNA is primarily expressed in the thymus and small intestine of mice, with low levels found in the liver, brain, testis, and effector T cells [81]. Our study is one of the few to show that subcutaneously administering LPS at a young age causes long-term changes in gene expression profiling in spleen tissue, with *Ccl25* being up-regulated in the treated mice. Meanwhile, [81] reported that LPS stimulates *Ccl25* expression in the spleen of mice in vivo. In the thymus, the CCR9 receptor is involved in T-cell development and trafficking [82]. Ccl25 is also produced by dendritic cells and epithelial cells in the thymus and possibly in the spleen [83]. *Ccl25*, which is expressed in the small intestinal epithelium, aids in the recruitment of T and B cells that express the CCR9 receptor [84]. According to Eksteen et al. [85], CCL25 was found in high concentrations in hepatic blood vessels in inflamed livers. The overexpression of *Ccl25* in spleen tissue is thought to play a similar role in the recruitment of T and B cells into the spleen. 

One of the up-regulated genes in the spleen of mice treated with subcutaneous LPS was *Cxcl5*. The protein encoded by *Cxcl5* (epithelial cell-derived neutrophil attractant) is a chemokine that has been demonstrated to attract neutrophils at the site of infection. In a study by Jeyaseelan et al. [86], mice were treated with aerosolized LPS to induce acute lung injury. The authors reported an overexpression of *Cxcl5*. Acute lung injury is characterized by an abnormally high neutrophil influx into the lungs, which has been linked to increased blood endotoxin concentrations as well as endothelial and epithelial damage in the lungs. Previous studies have indicated that FVB/N mice exhibit higher susceptibility to lung tumors while being comparatively less susceptible to liver tumors and lymphomas when compared to other mouse strains [87]. However, it is important to note that in our study, we employed the same genotype, ensuring that any observed results are primarily attributed to the effects of LPS treatment rather than the influence of the genotype. Although there are no data on the effects of LPS on *Cxcl5* in the spleen, *Cxcl5* overexpression in the spleen of mice in our experiment could be a response to subcutaneous LPS to attract neutrophils to the spleen. In this study, we did not collect data on the incidence of cancer, diabetes, and/or steatosis. Consequently, future studies are crucial to investigate and determine the potential role of LPS in the development and occurrence of these diseases.

Overall, the mechanism by which early-life subcutaneous administration of LPS for 6 weeks in female FVB/N mice caused long-term changes in gene expression of various chemokines and chemokine receptors in the liver and spleen is unknown; we believe that LPS caused epigenetic changes in the liver and spleen cells of the treated mice. This hypothesis is supported by recent studies using bovine cells treated with LPS and reporting that the latter induces DNA methylation. A study of bovine endometrial cells treated with LPS, for example, discovered that LPS induced DNA methylation patterns in bovine endometrial epithelial cells as well as activated proinflammatory mechanisms that disrupted immune balance and endometrial adhesion processes [88]. Another study discovered that low doses of bacterial LPS caused DNA hypomethylation in bovine mammary epithelial cells, while high doses caused hypermethylation [89]. 

## 5. Conclusions

In summary, our findings suggest that early-life (35-day old) exposure to chronic and continuous (for 6 weeks) endotoxemia may initiate molecular mechanisms that lead to long-term differential gene expression changes related to inflammation and adaptive immunity and may be involved in the pathobiology of a variety of chronic inflammatory diseases in the liver and spleen. Chronic endotoxemia may also contribute to hepatic steatosis and liver cancer in LPS-treated mice, according to our findings. Changes in gene expression related to energy metabolism, the suppression of TLR and immune responses, and the upregulation of genes related to B-cell and T-cell receptors, as well as chemokine signaling pathways, may all result from LPS treatment. The spleen plays a role in chronic endotoxemia response by removing LPS from circulation, establishing innate and adaptive immune responses, recruiting eosinophils and T cells, and promoting humoral immune responses. More research using a greater number of animals is needed to understand the long-term effects of early endotoxin exposure on gene expression in the liver, spleen, and possibly other organs.

## Figures and Tables

**Table 1:** List of differentially regulated genes in the LPS-treated group in the liver.

Gene Symbol	Description of the Genes	Fold-Change	*p*-Value
	Up-Regulated DE Genes in LPS vs. Saline		
*Pparg*	Peroxisome proliferator activated receptor gamma	4.18	0.02
*Frs3*	Fibroblast growth factor receptor substrate 3	2.65	0.0005
*Kras*	V-Ki-ras2 Kirsten rat sarcoma viral oncogene homolog	2.08	0.0008
*Raf1*	V-raf-leukemia viral oncogene 1	1.66	0.006
*Gsk3b*	Glycogen synthase kinase 3 beta	1.66	0.007
*Rras2*	Related RAS viral (r-ras) oncogene homolog 2	1.65	0.007
*Hk2*	Hexokinase 2	2.09	0.08
*Pik3r2*	Phosphatidylinositol 3-kinase, regulatory subunit, polypeptide 2 (p85 beta)	2.05	0.06
	**Down-Regulated DE genes in LPS vs. Saline**		
*Cxcl10*	Chemokine (C-X-C motif) ligand 10	−3.57	0.01
*Ptprf*	Protein tyrosine phosphatase, receptor type, F	−2.41	0.07
*Acaca*	Acetyl-Coenzyme A carboxylase alpha	−2.41	0.08
*Ccr5*	Chemokine (C-C motif) receptor 5	−2.2	0.06
*Irf3*	Interferon regulatory factor 3	−1.7	0.06
*Irf7*	Interferon regulatory factor 7	−1.7	0.06
*Lyz2*	Lysozyme 2	−2.2	0.06
*Mbl2*	Mannose-binding lectin (protein C) 2	−1.7	0.06
*Stat1*	Signal transducer and activator of transcription 1	−3.5	0.08

**Table 2:** List of differentially regulated genes in the LPS-treated group in the spleen.

Gene Symbol	Description of the Gene	Fold-Change	*p*-Value
	Up-Regulated DE Genes in LPS vs. Saline		
*Ccl25*	Chemokine (C-C motif) ligand 25	2.27	0.0002
*Il6*	Interleukin 6	2.89	0.0007
*Ccl17*	Chemokine (C-C motif) ligand 17	1.78	0.0009
*Pparg*	Peroxisome proliferating receptor gamma	5.81	0.001
*Ccl11*	Chemokine (C-C) ligand 11	2.29	0.001
*Prnd*	Prion protein 2 (dublet)	2.9	0.002
*Cxcl5*	Chemokine (C-X-C motif) ligand 5	1.77	0.006
*Il1a*	Interleukin 1a	1.8	0.011
*Cxcl11*	Chemokine (C-X-C motif) ligand 11	2.83	0.025
*Nos2*	Nitric oxide synthase 2	1.11	0.035
*Tlr4*	Toll like receptor 4	1.43	0.039
*Fcgr3*	Fc fragment of Ig, low affinity Il1b, receptor	18.23	0.045
	**Down-Regulated DE genes in LPS vs. Saline**		
*Fyn*	Fyn oncogene related to SRC, FGR YES	−4.41	0.002
*Grn*	Granulin	−2.19	0.003
*Lyz2*	Lysozyme 2	−11.02	0.005
*H2-k1*	Histocompatibility 2, K1, K region; similar to H-2K(d) antigen	−4.38	0.003
*Fcgr2b*	Fc receptor, IgG, low affinity IIb	−4.4	0.006
*Egr1*	Early growth response 1	−2.2	0.009
*Notch1*	Notch1	−2.21	0.010
*Rtp4*	Receptor (chemosensory) transporter protein4	−1.41	0.014
*Ifi27i2a*	Interferon, alpha-inducible protein 27 like 2A	−8.94	0.016
*Ache*	Acetylcholinesterase	−1.1	0.019
*Apoe*	Apolipoprotein E	−1.09	0.065
*Gbp4*	Guanylate binding protein 4	−2.2	0.072
*Atp1b1*	ATPase, Na^+^/K^+^ transporting, beta 1 polypeptide	−2.74	0.090

**Table 3:** Enriched pathway clusters determined by DAVID functional annotation clustering of up- regulated and down-regulated DE genes in CTR vs. LPS in mice liver.

Annotation Cluster 1: Enrichment Score: 2.67					
Category	Term	Genes	Count	*p*-Value	Benjamini
KEGG_PATHWAY	Hepatitis C	*Kras, Cxcl10, Gsk3b, Raf1*	4	5.90 × 10^−5^	5.60 × 10^−3^
KEGG_PATHWAY	Chemokine signaling pathway	*Kras, Cxcl10, Gsk3b, Raf1*	4	9.20 × 10^−5^	5.60 × 10^−3^
GOTERM_BP_DIRECT	Signal transduction	*Kras, Gsk3b, Pparg, Rras2, Raf1*	5	3.10 × 10^−4^	1.00 × 10^−1^
KEGG_PATHWAY	Endometrial cancer	*Kras, Gsk3b, Raf1*	3	4.00 × 10^−4^	1.40 × 10^−2^
KEGG_PATHWAY	Prolactin signaling pathway	*Kras, Gsk3b, Raf1*	3	6.50 × 10^−4^	1.40 × 10^−2^
KEGG_PATHWAY	EGFR tyrosine kinase inhibitor resistance	*Kras, Gsk3b, Raf1*	3	7.50 × 10^−4^	1.40 × 10^−2^
KEGG_PATHWAY	B-cell receptor signaling pathway	*Kras, Gsk3b, Raf1*	3	7.80 × 10^−4^	1.40 × 10^−2^
KEGG_PATHWAY	ErbB signaling pathway	*Kras, Gsk3b, Raf1*	3	8.40 × 10^−4^	1.40 × 10^−2^
KEGG_PATHWAY	Colorectal cancer	*Kras, Gsk3b, Raf1*	3	9.30 × 10^−4^	1.40 × 10^−2^
KEGG_PATHWAY	Prostate cancer	*Kras, Gsk3b, Raf1*	3	1.20 × 10^−3^	1.40 × 10^−2^
KEGG_PATHWAY	Melanogenesis	*Kras, Gsk3b, Raf1*	3	1.20 × 10^−3^	1.40 × 10^−2^
KEGG_PATHWAY	T cell receptor signaling pathway	*Kras, Gsk3b, Raf1*	3	1.30 × 10^−3^	1.40 × 10^−2^
KEGG_PATHWAY	Growth hormone synthesis, secretion and action	*Kras, Gsk3b, Raf1*	3	1.60 × 10^−3^	1.40 × 10^−2^
KEGG_PATHWAY	Thyroid hormone signaling pathway	*Kras, Gsk3b, Raf1*	3	1.70 × 10^−3^	1.40 × 10^−2^
KEGG_PATHWAY	Neurotrophin signaling pathway	*Kras, Gsk3b, Raf1*	3	1.70 × 10^−3^	1.40 × 10^−2^
KEGG_PATHWAY	Pathways in cancer	*Kras, Gsk3b, Pparg, Raf1*	4	2.00 × 10^−3^	1.40 × 10^−2^
KEGG_PATHWAY	Insulin signaling pathway	*Kras, Gsk3b, Raf1*	3	2.30 × 10^−3^	1.40 × 10^−2^
KEGG_PATHWAY	Signaling pathways regulating pluripotency of stem cells	*Kras, Gsk3b, Raf1*	3	2.30 × 10^−3^	1.40 × 10^−2^
KEGG_PATHWAY	Breast cancer	*Kras, Gsk3b, Raf1*	3	2.60 × 10^−3^	1.40 × 10^−2^
KEGG_PATHWAY	Gastric cancer	*Kras, Gsk3b, Raf1*	3	2.70 × 10^−3^	1.40 × 10^−2^
KEGG_PATHWAY	mTOR signaling pathway	*Kras, Gsk3b, Raf1*	3	2.90 × 10^−3^	1.50 × 10^−2^
KEGG_PATHWAY	Hepatocellular carcinoma	*Kras, Gsk3b, Raf1*	3	3.60 × 10^−3^	1.70 × 10^−2^
KEGG_PATHWAY	Axon guidance	*Kras, Gsk3b, Raf1*	3	3.90 × 10^−3^	1.80 × 10^−2^
KEGG_PATHWAY	Kaposi sarcoma-associated herpesvirus infection	*Kras, Gsk3b, Raf1*	3	5.90 × 10^−3^	2.40 × 10^−2^
KEGG_PATHWAY	Human cytomegalovirus infection	*Kras, Gsk3b, Raf1*	3	7.60 × 10^−3^	2.80 × 10^−2^
GOTERM_MF_DIRECT	nucleotide binding	*Kras, Gsk3b, Rras2, Raf1*	4	1.30 × 10^−2^	5.10 × 10^−1^
KEGG_PATHWAY	PI3K-Akt signaling pathway	*Kras, Gsk3b, Raf1*	3	1.50 × 10^−2^	5.00 × 10^−2^
KEGG_PATHWAY	Human papillomavirus infection	*Kras, Gsk3b, Raf1*	3	1.50 × 10^−2^	5.00 × 10^−2^
KEGG_PATHWAY	Alzheimer’s disease	*Kras, Gsk3b, Raf1*	3	1.70 × 10^−2^	5.50 × 10^−2^
KEGG_PATHWAY	Pathways of neurodegeneration—multiple diseases	*Kras, Gsk3b, Raf1*	3	2.50 × 10^−2^	7.50 × 10^−2^
GOTERM_MF_DIRECT	protein binding	*Kras, Gsk3b, Pparg, Rras2, Raf1*	5	6.30 × 10^−2^	8.70 × 10^−1^

Category: original database/resource where the terms originate; Term: enriched terms associated with our gene list; Count: genes involved in the term; *p*-value: Benjamini adjusted *p*- value. GOTERM: Gene Ontology Term; BP: biological process; MF: molecular function.

**Table 4:** Most enriched pathway clusters as determined by DAVID functional annotation clustering of up-regulated and down- regulated DE genes in CTR vs. LPS in mouse spleens.

Annotation Cluster 1: Enrichment Score: 4.25					
Category	Term	Genes	Count	*p*-Value	Benjamini
GOTERM_BP_DIRECT	Inflammatory response	*Ccl11, Ccl17, Ccl25, Cxcl11, Cxcl5, Il1a, Il6, Nos2, Pparg, Tlr4*	10	5.80 × 10^−11^	4.20 × 10^−8^
GOTERM_BP_DIRECT	Neutrophil chemotaxis	*Fgr, Ccl11, Ccl17, Ccl25, Cscl11, Cxcl5*	6	1.40 × 10^−8^	5.20 × 10^−6^
GOTERM_BP_DIRECT	positive regulation of ERK1 and ERK2 cascade	*Ccl11, Ccl17, Ccl25, Il1a, Il6, Notch1, Tlr4*	7	9.50 × 10^−8^	2.30 × 10^−5^
GOTERM_MF_DIRECT	Cytokine activity	*Ccl11, Ccl17, Ccl25, Cxcl5, Grn, Il1a, Il6*	7	1.30 × 10^−7^	9.30 × 10^−6^
GOTERM_MF_DIRECT	Chemokine activity	*Ccl11, Ccl17, Ccl25, Cxcl11, Cxcl5*	5	1.30 × 10^−7^	9.30 × 10^−6^
GOTERM_BP_DIRECT	Chemokine-mediated signaling pathway	*Ccl11, Ccl17, Ccl25, Cxcl11, Cxcl5*	5	2.20 × 10^−7^	4.10 × 10^−5^
GOTERM_BP_DIRECT	Immune response	*Ccl11, Ccl25, CxCl11, Cxcl5, Il1a, H2-k1, Tlr4*	8	3.20 × 10^−7^	4.60 × 10^−4^
KEGG_PATHWAY	Viral protein interaction with cytokine and cytokine receptor	*Ccl11, Ccl17, Ccl25, Cxcl11, Cxcl5, Il6*	6	6.60 × 10^−7^	7.30 × 10^−4^
REACTOME_PATHWAY	Chemokine receptors bind chemokines	*Ccl11, Ccl17, Ccl25, Cxcl11, Cxcl5*	5	2.40 × 10^−6^	3.30 × 10^−4^
GOTERM_BP_DIRECT	Cellular response to interferon-gamma	*Ccl11, Ccl17, Ccl25, Nos2, Tlr4*	5	4.10 × 10^−6^	4.90 × 10^−4^
GOTERM_BP_DIRECT	Chemotaxis	*Ccl11, Ccl17, Ccl25, Cxcl11, Cxcl5*	5	9.10 × 10^−6^	8.30 × 10^−4^
KEGG_PATHWAY	Cytokine–cytokine receptor interaction	*Ccl11, Ccl17, Ccl25, Cxcl11, Cxcl5, Il1a, Il6*	7	1.00 × 10^−5^	4.00 × 10^−4^
GOTERM_BP_DIRECT	Antimicrobial humoral immune response mediated by antimicrobial peptide	*Ccl11, Ccl17, Ccl25, Cxcl11, Cxcl5*	5	1.10 × 10^−5^	8.50 × 10^−4^
GOTERM_BP_DIRECT	Killing of cells of other organisms	*Ccl11, Ccl17, Ccl25, Lyz2*	4	1.90 × 10^−5^	1.40 × 10^−3^
GOTERM_BP_DIRECT	Cellular response to interleukin-1	*Ccl11, Ccl17, Ccl25, Il6*	4	7.20 × 10^−5^	4.80 × 10^−3^
REACTOME_PATHWAY	Peptide ligand-binding receptors	*Ccl11, Ccl17, Ccl25, Cxcl11, Cxcl5*	5	3.20 × 10^−5^	2.10 × 10^−2^
GOTERM_MF_DIRECT	CCR chemokine receptor binding	*Ccl11, Ccl17, Ccl25*	3	3.50 × 10^−4^	1.70 × 10^−2^
KEGG_PATHWAY	Chemokine signaling pathway	*Ccl11, Ccl17, Ccl25, Cxcl11, Cxcl5*	5	3.80 × 10^−4^	6.10 × 10^−2^
GOTERM_BP_DIRECT	Cellular response to tumor necrosis factor	*Ccl11, Ccl17, Ccl25, Il6*	4	4.00 × 10^−4^	1.60 × 10^−2^
GOTERM_BP_DIRECT	Lymphocyte chemotaxis	*Ccl11, Ccl17, Ccl25*	3	4.10 × 10^−4^	1.60 × 10^−2^
GOTERM_BP_DIRECT	Monocyte chemotaxis	*Ccl11, Ccl17, Ccl25*	3	6.20 × 10^−4^	2.20 × 10^−2^
KEGG_PATHWAY	IL-17 signaling pathway	*Ccl11, Ccl17, Ccl25, Il6*	4	6.50 × 10^−4^	8.10 × 10^−3^
REACTOME_PATHWAY	Class A/1 (Rhodopsin-like receptors)	*Ccl11, Ccl17, Ccl25, Cxcl11, Cxcl5*	5	2.10 × 10^−3^	9.30 × 10^−2^
GOTERM_BP_DIRECT	Cell chemotaxis	*Ccl17, Ccl25, Cxcl11*	3	3.50 × 10^−3^	7.50 × 10^−2^
REACTOME_PATHWAY	GPCR ligand binding	*Ccl11, Ccl17, Ccl25, Cxcl11, Cxcl5*	5	5.20 × 10^−3^	1.20 × 10^−1^
REACTOME_PATHWAY	G alpha (i) signaling events	*Ccl11, Ccl25, Cxcl11, Cxcl5*	4	1.30 × 10^−2^	2.50 × 10^−1^
GOTERM_BP_DIRECT	Positive regulation of GTPase activity	*Ccl11, Ccl17, Ccl25*	3	1.60 × 10^−2^	2.20 × 10^−1^
REACTOME_PATHWAY	Signaling by GPCR	*Ccl11, Ccl17, Ccl25, Cxcl11, Cxcl5*	5	2.20 × 10^−2^	3.60 × 10^−1^
REACTOME_PATHWAY	Signal transduction	*Fyn, Ccl11, Ccl17, Ccl25, Cxcl11, Cxcl5, Il6, Notch1*	8	8.20 × 10^−2^	8.90 × 10^−1^
REACTOME_PATHWAY	GPCR downstream signaling	*Ccl11, Ccl25, Cxcl11, Cxcl5*	4	8.50 × 10^−2^	8.90 × 10^−1^
GOTERM_BP_DIRECT	G-protein coupled receptor signaling pathway	*Ccl11, Ccl17, Ccl25*	3	5.20 × 10^−1^	1.00 × 10^−1^
**Annotation Cluster 2: Enrichment Score: 3.07**					
GOTERM_BP_DIRECT	Cellular response to lipopolysaccharide	*Cxcl11, Cxcl5, Il1a, Il6, Nos2, Tlr4*	6	8.90 × 10^−6^	8.30 × 10^−4^
KEGG_PATHWAY	Pertussis	*Cxcl5, Il1a, Il6, Nos2, Tlr4*	5	1.10 × 10^−5^	4.00 × 10^−4^
KEGG_PATHWAY	Tuberculosis	*Fcgr3, Fcgr2b, Il1a, Nos2, Tlr4*	6	1.50 × 10^−5^	4.30 × 10^−4^
GOTERM_BP_DIRECT	Positive regulation of interleukin-6 production	*Il1a, Il6, Nos2, Tlr4*	4	1.80 × 10^−4^	1.00 × 10^−2^
GOTERM_BP_DIRECT	Positive regulation of apoptotic process	*Il6, Nos2, Notch1, Pparg, Tlr4*	5	5.00 × 10^−4^	1.80 × 10^−2^
GOTERM_BP_DIRECT	Positive regulation of interleukin-8 production	*Il6, Nos2, Tlr4*	3	1.60 × 10^−3^	3.90 × 10^−2^
GOTERM_BP_DIRECT	Signal transduction	*Ccl17, Cxcl11, Il6, Nos2, Pparg, Tlr4*	6	1.00 × 10^−2^	1.60 × 10^−1^
KEGG_PATHWAY	Toll-like receptor signaling pathway	*Cxcl11, Il6, Tlr4*	3	1.50 × 10^−2^	1.00 × 10^−1^
KEGG_PATHWAY	Chagas disease	*Il6, Nos2, Tlr4*	3	1.60 × 10^−2^	1.00 × 10^−1^
KEGG_PATHWAY	Amoebiasis	*Il6, Nos2, Tlr4*	3	1.70 × 10^−2^	1.10 × 10^−1^
KEGG_PATHWAY	HIF-1 signaling pathway	*Il6, Nos2, Tlr4*	3	1.90 × 10^−2^	1.10 × 10^−1^

Category: original database/resource where the terms origin; Term: enriched terms associated with our gene list; Count: genes involved in the term; *p*-value: Benjamini adjusted *p*-value GOTERM: Gene Ontology Term; BP: biological process; MF: molecular function.

## Data Availability

All data analyzed during this study are included in this published article.

## Supplementary Materials

The following supporting information can be downloaded at: https://www.mdpi.com/article/10.3390/vetsci10070445/s1, Figure S1: The weight of the mice treated with lipopolysaccharide (LPS) and control (CON) during the experimental period.
