# Supplementary material for: Early-Life Exposure to Lipopolysaccharide Induces Persistent Changes in Gene Expression Profiles in the Liver and Spleen of Female FVB/N Mice
Source: Vet Sci. 2023 Jul 8;10(7):445. doi: 10.3390/vetsci10070445 (PMC10384579; doi:10.3390/vetsci10070445)

Figure S1. The weight of the mice treated with lipopolysaccharide (LPS) and control (CON) during the experimental period.

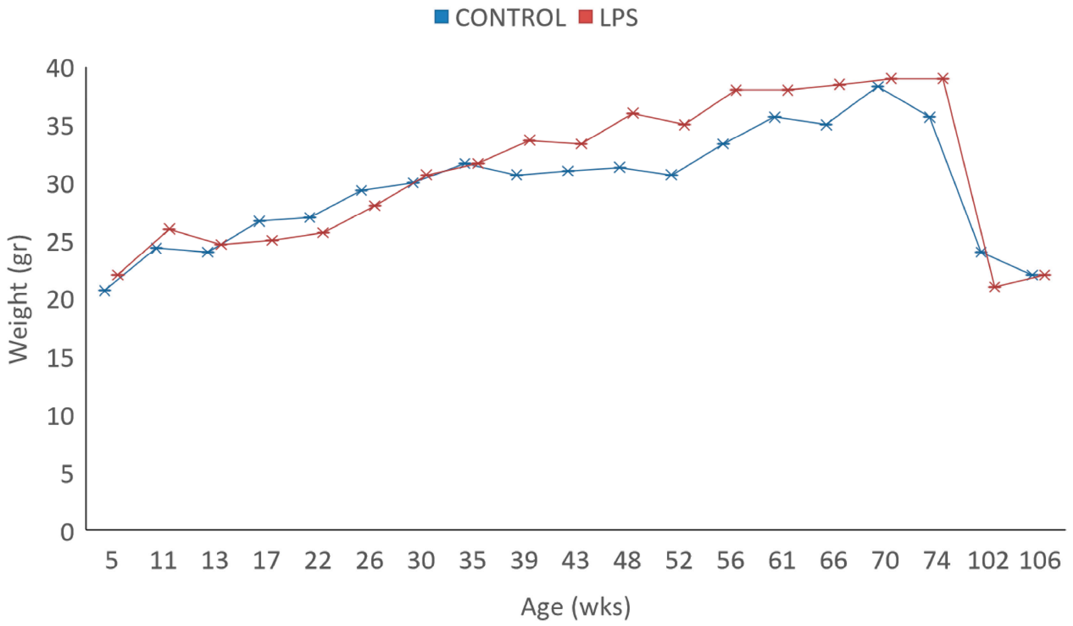

Supplement: Supplementary file 1 [file vetsci-10-00445-s001.zip › vetsci-2436206-supplementary.pdf]
